# Proposal of *Thermoactinomyces mirandus* sp. nov., a filamentous, anaerobic bacterium isolated from a biogas plant

**DOI:** 10.1007/s10482-020-01497-0

**Published:** 2020-11-19

**Authors:** Mira Mutschlechner, Nina Lackner, Rudolf Markt, Willi Salvenmoser, Christopher A. Dunlap, Andreas O. Wagner

**Affiliations:** 1 Department of Microbiology, Universität Innsbruck, Technikerstraße 25d, 6020 Innsbruck, Austria; 2 Department of Zoology, Universität Innsbruck, Technikerstraße 25, 6020 Innsbruck, Austria; 3 Crop Bioprotection Research Unit, Agricultural Research Service, US Department of Agriculture, National Center for Agricultural Utilization Research, Peoria, IL 61604 USA

**Keywords:** Hydrogen production, Thermophilic, NiFe, Hydrogenase, Hyp, Novobiocin, Thermoactinomycetaceae, Fermentation

## Abstract

We isolated a filamentous, thermophilic, and first anaerobic representative of the genus *Thermoactinomyces*, designated strain AMNI-1^T^, from a biogas plant in Tyrol, Austria and report the results of a phenotypic, genetic, and phylogenetic investigation. Strain AMNI-1^T^ was observed to form a white branching mycelium that aggregates into pellets when grown in liquid medium. Cells could primarily utilize lactose, glucose, and mannose as carbon and energy sources, with acetate accelerating and yeast extract being mandatory for growth. The optimum growth temperature and pH turned out to be 55 °C and pH 7.0, respectively, with an optimum NaCl concentration of 0–2% (w/v). 16S rRNA gene sequence comparison indicated that the genetic relatedness between strain AMNI-1^T^ and *Thermoactinomyces intermedius, Thermoactinomyces khenchelensis,* and *Thermoactinomyces vulgaris* was less than 97%. The G + C content of the genomic DNA was 44.7 mol%. The data obtained suggest that the isolate represents a novel and first anaerobic species of the genus *Thermoactinomyces*, for which the name *Thermoactinomyces mirandus* is proposed. The type strain is AMNI-1^T^ (= DSM 110094^T^ = LMG 31503^T^). The description of the genus *Thermoactinomyces* is emended accordingly.

## Introduction

The genus *Thermoactinomyces* within the family Thermoactinomycetaceae was initially described by Tsilinsky (1899), with the first known representative *Thermoactinomyces vulgaris* being isolated from decaying straw and manure. Since then, several species of *Thermoactinomyces* have been isolated from a variety of habitats (Bezuidt et al. 2016). In 2005, several former *Thermoactinomyces* species were reclassified in new genera *Laceyella*, *Thermoflavimicrobium* and *Seinonella* (Yoon et al. 2005). Currently, the genus comprises four validly described species including *Thermoactinomyces vulgaris* (Tsilinsky 1899), *Thermoactinomyces intermedius* (Kurup et al. 1980), *Thermoactinomyces daqus* (Yao et al. 2014), and *Thermoactinomyces khenchelensis* (Mokrane et al. 2016). All these species are aerobic, Gram-positive, chemoorganotrophic, filamentous, thermophilic bacteria with a G + C content in the range of 48.0–49.1 mol% (Lacey and Cross 1989). Aerial mycelium is abundant and white. Well-developed, branched and septate substrate mycelium is formed.

Here we describe strain AMNI-1^T^ that was isolated from a biogas plant. Although sharing certain common phenotypic and genotypic characteristics to members of the genus *Thermoactinomyces* including filamentous growth, resistance to novobiocin, and 16S rRNA gene sequence similarity, the novel strain AMNI-1^T^ requires anaerobic conditions for its fermentative metabolism in contrast to its closest relatives. Therefore, we propose that strain AMNI-1^T^ should be classified as a novel species and first anaerobic representative of the genus *Thermoactinomyces*. As a consequence, the genus should be emended to include anaerobic members with a fermentative, acid and hydrogen producing metabolism.

## Materials and methods

### Isolation and ecology

Strain AMNI-1^T^ was isolated from a biogas plant located in Roppen (Tyrol, Austria, 47.230 N 10.833 E) in 2015. The anaerobic digestion plant represents a 750.000-L plug-flow digester that is operated under thermophilic conditions and designed to treat separately gathered organic fractions of household wastes (Wagner et al. 2018). For a description of running parameters and physical–chemical properties of the reactor please refer to (Illmer et al. 2009). Fermenter sludge samples were withdrawn from the inlet of the biogas reactor via a sampling port by removing approx. 1 kg of sludge, diluted and incubated under thermophilic conditions as described previously (Wagner et al. 2014) and subsequently used as inoculum by multiple transfers into deep agar shakes. One isolate, which we designated as AMNI-1^T^, was selected for further characterization. After isolation, strain AMNI-1^T^ was repeatedly transferred in fresh liquid media as described below by particularly selecting visible white filaments.

### Physiological characterization

The medium used for long-term maintenance of the pure culture contained (in g L^−1^): 0.35 K_2_HPO_4_, 0.23 KH_2_PO_4_, 0.5 MgSO_4_·7 H_2_O, 0.05 CaCl_2_·2 H_2_O, 2.25 NaCl, 0.002 FeSO_4_·7 H_2_O, 0.5 NH_4_Cl, 1.0 yeast extract, 2.0 lactose, 0.5 sodium acetate, and 0.5 l-cysteine HCl. After solving all ingredients, the medium was amended with 1.0 mL SL-10 (Medium 120, German Collection of Microorganisms and Cell Cultures GmbH) and 1 mL resazurin (0.1% w/v), with the pH being adjusted to 6.8, if not indicated otherwise. Aliquots of 50 mL were filled in 120 mL serum flasks and heated in a water bath for 20 min to reduce the solubility of O_2_. The headspaces were immediately flushed with N_2_, closed with butyl rubber septa, and sealed with aluminium caps. Before autoclaving, 0.5 mL Na_2_S (12.0 g L^−1^) together with NaHCO_3_ (50.0 g L^−1^) solution was added to each flask to decrease the redox potential and as buffering agent (Wagner et al. 2019). The medium was inoculated with 1 mL of active cultures (less than 36 h of age as the viability decreases with time) and incubated at 55 °C, if not indicated otherwise. For single parameter variation studies, the concentration of yeast extract was reduced to 0.1 g L^−1^.

Growth was determined after 3 days of incubation via visible growth and evaluation of H_2_ and CO_2_ production via gas chromatography by applying a thermal conductivity detector as described previously (Wagner et al. 2011). For positive growth, twice the mean of the controls (containing only 0.1 g L^−1^ as sole carbon source) was set as threshold for H_2_ and CO_2_ production. The fermentation products were detected and quantified using high performance liquid chromatography according to Wagner et al. (2017). The pH was measured with MColorpHast™ indicator stripes (Merck Millipore, Germany). Regarding chemotaxonomic characterisation, fatty acid methyl ester (FAME) profiling as proof of relationship seemed unreasonable due to the anaerobic nature of the strain in contrast to its aerobic nearest neighbours.

The temperature range for growth was evaluated by incubating the isolate at 25–65 °C (25, 30, 37, 45, 50, 55, 60, and 65 °C for 7 days). Growth was further tested at different initial pH values ranging from pH 5.0 to 9.0 (by adjusting with 1.0 M HCl and 1.0 M NaOH, respectively) and NaCl concentrations of 0, 0.225, 1.0, 2.0, 5.0, and 10.0% (w/v). Antibiotic testing of the strain was performed in the presence of novobiocin, streptomycin, ampicillin, gentamycin, chloramphenicol, tetracycline, and erythromycin (5 µg mL^−1^ each). Carbon sources were tested at a concentration of 0.8 g C L^−1^ (please see below). Aerobic growth was tested in the same medium by enriching the headspace with varying concentrations of O_2_.

### Phenotypic characterization

The morphological characteristics of strain AMNI-1^T^ were observed by light microscopy (Nikon Eclipse) as well as scanning (SEM) and transmission electron microscopy (TEM) (Zeiss, Germany) after incubation of 4 and 35 days. For SEM, samples were fixed with 2.5% glutaraldehyde in 0.1 M cacodylate buffer, washed in buffer and postfixed in 1% osmium tetroxide in 0.05 M cacodylate buffer. After washing with buffer, samples were dehydrated with an increasing ethanol series and critical point dried with an EMS 850 CPD (Electron Microscopy services, Germany). After mounting, 20 nm gold were sputtered onto the samples with a CCD-10 sputter coater (Safematic, Switzerland) and examined with a Zeiss DSM950 SEM (Zeiss, Germany). Images were taken with a Pentax digital camera and a PK_Tether 0.7.0 free software. For TEM, specimens were fixed with 2.5% glutaraldehyde in culture medium for 1 h at 4 °C, washed in 0.1 M cacodylate buffer and postfixed with 1% osmium tetroxide in 0.05 M cacodylate buffer for 1 h. Stain AMNI-1^T^ was dehydrated in an increasing acetone series and embedded into EMBed 812 epoxy resin. Sections were cut with a diamond knife (Diatome, Switzerland) on an Ultracut UCT ultramicrotome (Leica, Vienna), stained with lead citrate and examined with a Zeiss Libra 120 TEM (Zeiss, Germany). Images were taken with Image SP software and a high-speed 2 × 2 k camera (Tröndle, Germany).

### Phylogenetic analyses

The genomic DNA was extracted using a NucleoSpin^®^ Soil kit (Macherey–Nagel, Germany) according to the manufacturers protocol. Initially, the 16S rRNA gene was amplified using the universal primers 27f and 1492r (Heuer et al. 1997). Sanger sequencing was performed at Eurofins Genomics (Germany). Sequencing data were deposited in GenBank (accession number: MN148883). Subsequently, the genome of the strain and two close relatives (*Thermoactinomyces vulgaris* KACC 12356^T^ and *Thermoactinomyces intermedius* NRRL B-16979^T^) were sequenced to definitively confirm the taxonomy of the strain. The genomic DNA was prepared for sequencing using Nextera XT library preparation kit following the manufacturer’s suggested protocols. The prepared libraries were sequenced using a MiSeq DNA sequencer with the MiSeq V3 2x300 sequencing kit. The resulting reads were quality trimmed to the 95% confidence level. The draft genome was assembled using CLCbio Genomics Workbench 20.0 (Qiagen Inc, Cambridge, MA) using default parameters. The sequences for the subject strain, AMNI-1^T^ were deposited in NCBI Genbank under accession numbers JACEOL010000000 and a full length 16S rRNA was deposited under GenBank accession number JACEOL010000098. The sequences for *T. vulgaris* KACC 12356^T^ and *T. intermedius* NRRL B-16979^T^) were submitted to NCBI Genbank under accession numbers JACEOK010000000 and JACETT010000000, respectively.

The 16S rRNA phylogeny of the strain was performed using the genome derived 16S rRNA gene. Close relatives were identified using EZBioCloud 16S rRNA database (Yoon et al. 2017) and alignments were made with MEGA X. The Maximum-Likelihood tree was determined using the Tamura-Nei model (gamma distributed with invariant sites) based on model testing under MEGA X (Kumar et al. 2018). *Aneurinibacillus aneurinilyticus* was selected as an outgroup. Measures of bootstrap support for internal branches were obtained from 1500 pseudoreplicates. Additionally, a 92 gene nucleotide phylogeny was performed using UBCG software with default parameters using FastTree (Na et al. 2018). In addition to the genomes of all available type strains in the area, all genomes from representative strains from *Thermoactinomyces* and *Laceyella* genera were included. The average nucleotide identity (ANI) was determined using OrthoANI using default parameters on the website (Lee et al. 2016).

## Results and discussion

### Physiological and morphological characterization

**Fig. 1:**
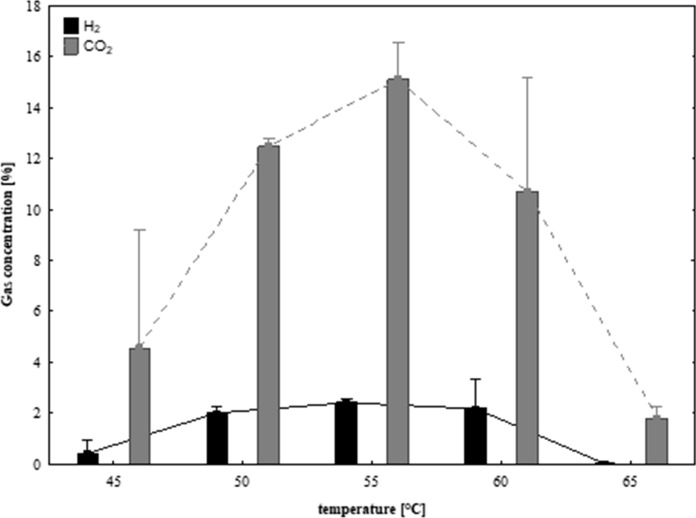
H_2_ and CO_2_ concentration quantified via gas chromatography (GC-TCD) in serum flasks with strain AMNI-1^T^ at different incubation temperatures. Results are given as mean (± SD), n = 3

Results of the physiological characterization are summarized in Table 1. Strain AMNI-1^T^ grew well on lactose, glucose, mannose, or lactose/acetate. Poor growth was observed when yeast extract or meat extract were the main carbon sources. Small amendments of yeast extract; however, were mandatory for growth on all substrates. Further, 1.0 g acetate L^−1^ accelerated growth. Substrates that could not be utilized included glycine, alanine, sucrose, methanol, ethanol, starch, peptone, lactate, and H_2_/CO_2_. In the presence of 0.1 g L^−1^ yeast extract, the end products of lactose fermentation were lactate, acetate, ethanol, H_2_ and CO_2_. Strain AMNI-1^T^ showed growth at temperatures ranging from 45 to 60 °C (Fig. 1) and at pH-values ranging from 5.0 to 9.0, optimal growth occurred at 55 °C and pH 7.0, respectively. The optimal NaCl concentration turned out to be in the range of 0–2.0% (w/v). The study of antibiotic susceptibility showed that strain AMNI-1^T^ was sensitive to a wide range of antibiotics including chloramphenicol, tetracycline, ampicillin, streptomycin, gentamycin, and erythromycin but not to novobiocin, which is a common characteristic feature of *Thermoactinomyces* species. Strain AMNI-1^T^ was unable to grow under microaerophilic and aerobic conditions. Once growth occurred, H_2_ production as well as increased CO_2_ concentrations were detectable (Fig. 1).

**Table 1:** Differential characteristics of strain AMNI-1^T^ and type strains of the genera *Thermoactinomyces* with an identity > 96%

Characteristics	1	2	3	4
Utilization of				
Glycine	−	−	nd	−
Glucose	+	−	+	−
Lactose	+	−	−	+
Starch	−	nd	nd	−
Resistance to novobiocin	+	+	+	+
Growth in the presence of 5% NaCl (w/v)	−	nd	+	−
Temperature range (°C)	45–60	37–65	37–55	45–60
G + C content (mol%)	44.7	48	nd	48
pH range	5.0–9.0	5.0–8.0	7.0–9.0	5.0–8.0
O_2_ requirements	Anaerobic	Aerobic	Aerobic	Aerobic
Endospores	Not observed	+	+	+

Strains: *1*, AMNI-1^T^; *2*, *T. intermedius* DSM 43846^T^; *3*, *T. khenchelensis* DSM 45951^T^; *4*, *T. vulgaris* DSM 43016^T^. Data on strain AMNI-1^T^ were determined in this study; data on 2, 3, and 4 were obtained from Kurup et al. (1980), Mokrane et al. (2016), and Tsilinsky (1899). +, positive; −, negative; *nd* not determined

**Fig. 2:**
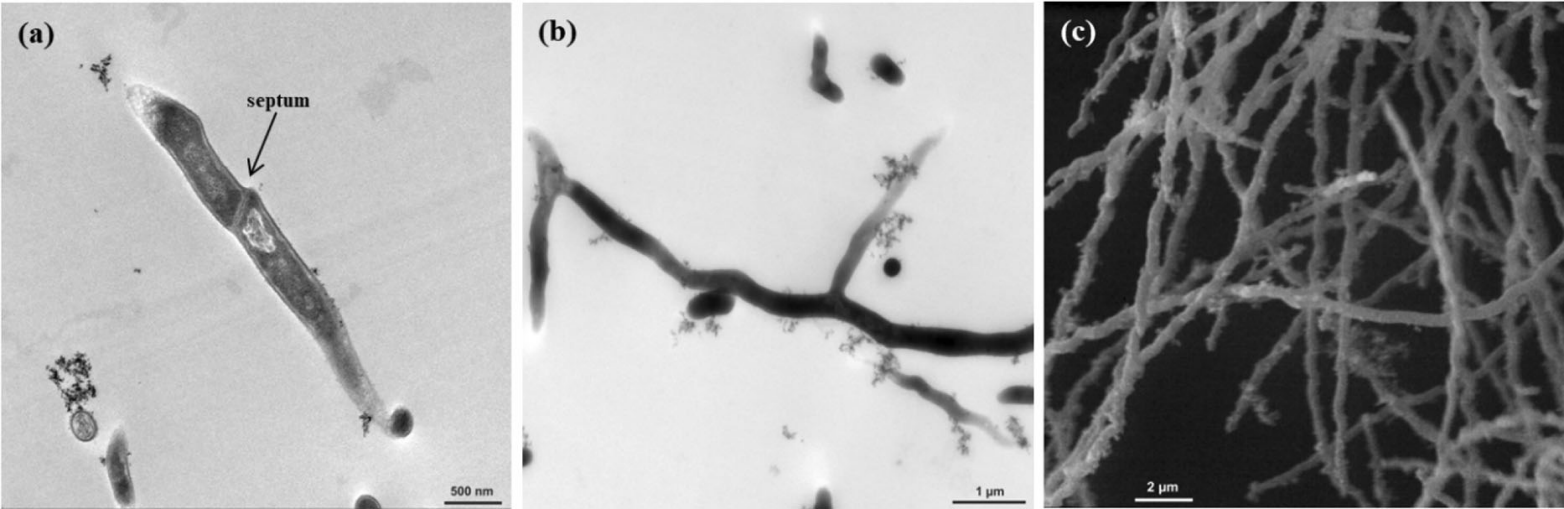
Transmission electron microscopy of strain AMNI-1^T^ after 4 days (**a**) and 35 days (**b**). Scanning electron microscopy of strain AMNI-1^T^ after 4 days (**c**)

Irrespective of shaking, strain AMNI-1^T^ formed white cloudy aggregates being able to float during phases of high gas production that were macroscopically visible. Light microscopy and SEM/TEM revealed hyphae like structures of approximately 0.3–0.5 µm diameter. The mycelium consisted of branched, septate, and corrugated filamentous structures (Fig. 2a–c).

The cell wall contains a meso-diaminopimelate peptidyl-glycan linkage based on comparisons of the peptidyl glycan ligase (MBA4601166.1) found in the subject strain and closely related types strains with verified linkages. Comparisons of cell wall fatty acids with closely related types strains was not performed, since suitable common growth conditions (aerobic vs. anaerobic) could not be identified. Fatty acids have not been used as distinguishing phenotypes in the taxonomy of *Thermoactinomyces* species.

### Phylogenetic interference and genome features

**Fig. 3:**
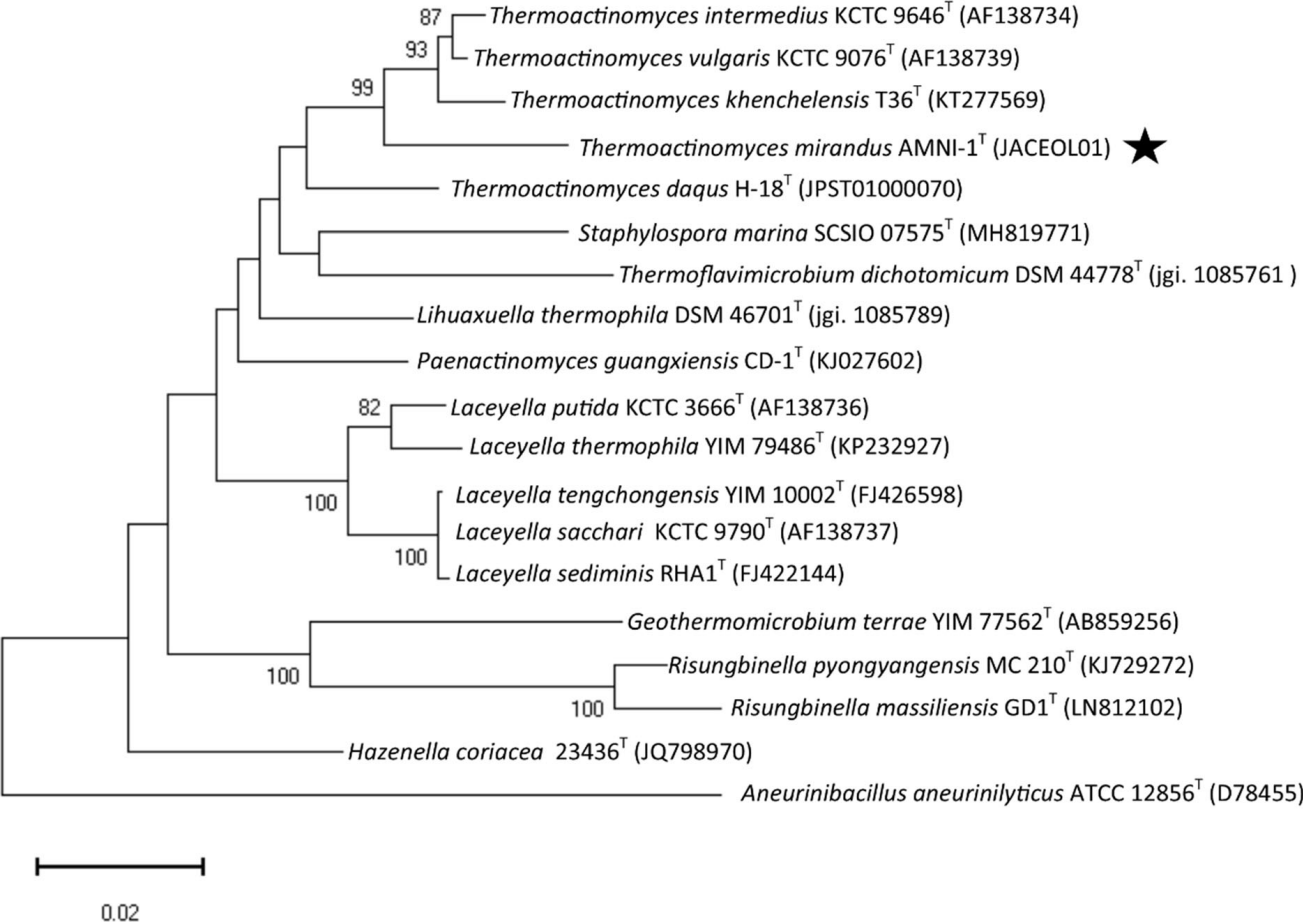
Phylogenetic tree showing the relationships of strain AMNI-1^T^ to members of related species. The tree was based on 16S rRNA gene sequences obtained from EzBioCloud reference database. Clustering was performed with maximum likelihood using the Tamura-Nei substitution model as determined through model testing under MEGA X. Bootstrap support was calculated based on 1500 pseudoreplicates and values below 70% are removed. Bar represents expected numbers of substitutions per nucleotide position

**Fig. 4:**
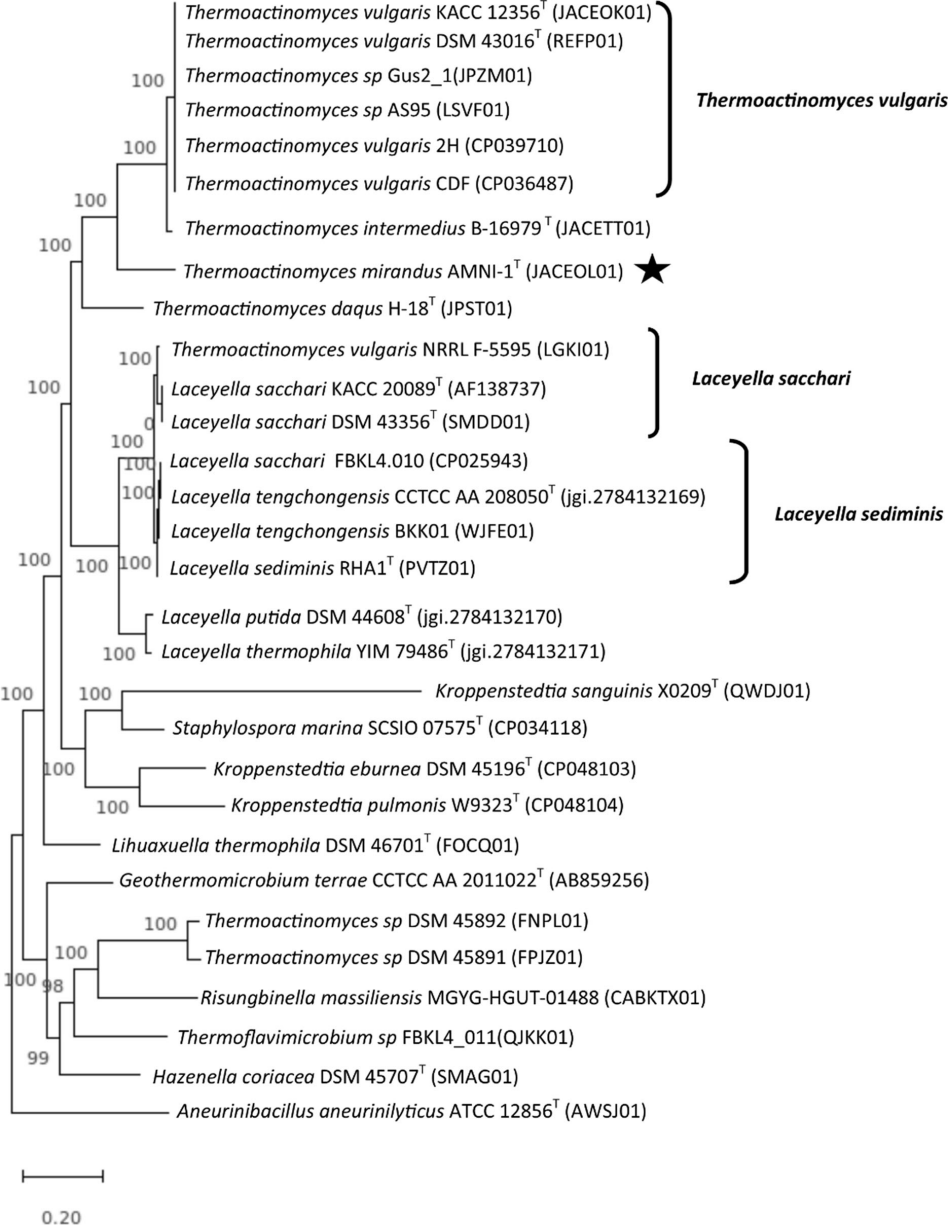
Genome-based phylogenetic tree showing the relationships of strain AMNI-1^T^ to members of related species. The tree was based on 92 genes in the up-to-date bacterial core genome (UBCG) (Na et al. 2018). The maximum likelihood tree was produced by FastTree under the default parameters in the UBCG pipeline. Bar represents expected numbers of substitutions per nucleotide position

**Fig. 5:**
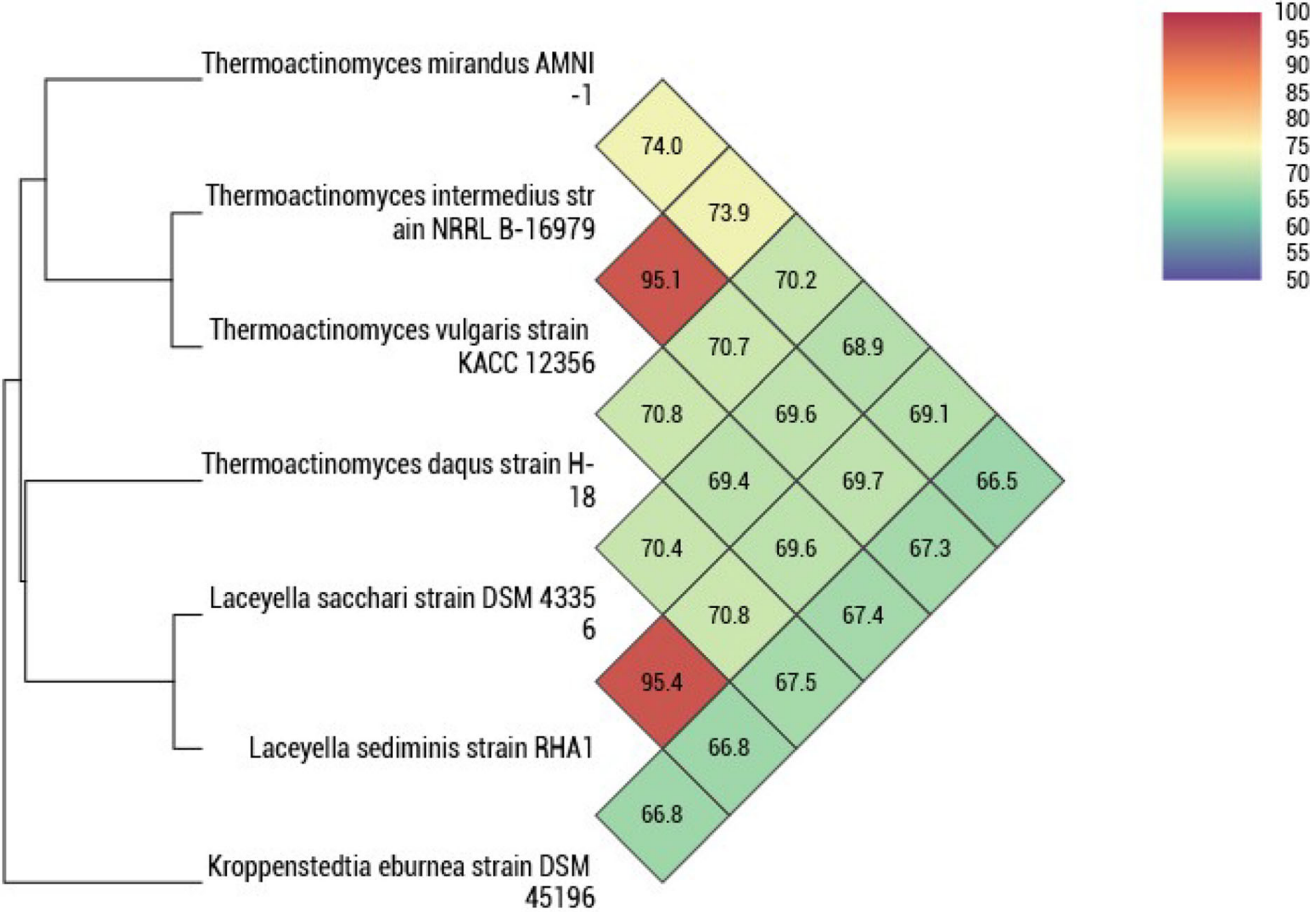
Average nucleotide identity analysis of closely related type strains

The 16S rRNA phylogeny has low bootstrap support for many of the branches (Fig. 3). While the UBCG 92 gene tree has high support for all branches (Fig. 4). There are some incongruences between the 16S rRNA and UBCG tree in the areas of the 16S rRNA gene tree with low bootstrap support. The inclusion of the representative (non-type) genomes showed some inconsistencies in the taxonomy they were accessioned under. Two strains (Genbank accession#: JPZM01 and LSVF01) accessioned under *Thermoactinomyes* sp. should be *T. vulgaris*. Two strains (Genbank accession#: FNLP01 and FNJZ01) accessioned under *Thermoactinomyes* sp. should be *Risungbinella* sp. Strain NRRL F-5595 (Genbank accession#: LGKI01) accessioned under *T. vulgaris* sp. should be *Laceyella sacchari*. Our results are also consistent with *Laceyella tengchongensis* being a later heterotypic synonym of *Laceyella sedeminis* as recently reported (Jiang et al. 2019). No public genomes were found to be conspecific with the subject strain. Average nucleotide identity analysis of the type strains in this area confirmed strain AMNI-1^T^ was a novel species with the closest type strain having an ANI of 74.0 (Fig. 5). The G + C content of the DNA was determined from the genome data. The DNA G + C content of strain AMNI-1^T^ was 44.7 mol%, which is lower compared with its closest phylogenetic relatives (Table 1).

After quality trimming, Sanger sequencing of the 16S rRNA gene yielded a consensus sequence of 1365 bp, which was aligned to the 16S rRNA database. The maximum likelihood analysis of the resulting alignment (1352 bp) placed strain AMNI-1^T^ in the family Thermoactinomycetaceae, with the strain forming a common clade with species of the genus *Thermoactinomyces*. The closest relatives are *T. intermedius, T. khenchelensis,* and *T. vulgaris*, with sequence identities of 96.3%, 96.2%, and 96.2%, respectively. Based on its phenotypic and genotypic characteristics, strain AMNI-1^T^ should be described as a novel species belonging to the *Thermoactinomyces* genus, for which the name *Thermoactinomyces mirandus* sp. nov. is proposed. The type strain is AMNI-1^T^ (= DSM 110094^T^ = LMG 31503^T^).

Genome sequencing showed that strain AMNI-1^T^ encodes an 11 gene cluster for a structural [NiFe]-hydrogenase and its assembly as well as maturation (locus_tags: H2C83.05685..H2C83.05735), which was rather surprising as none of these particular genes have been identified in any other, already sequenced species of the family Thermoactinomycetaceae. [NiFe] hydrogenases represent a diverse group of metalloenzymes that catalyse reversible hydrogen production/consumption (H_2_ < − > 2H + + 2e −) (Teng et al. 2019; Marshall et al. 2012), thus coinciding with our GC analyses revealing hydrogen formation (Fig. 1). The biosynthesis/maturation of these enzymes constitutes a complex process that involves six Hyp proteins, all of which have been found in the present investigation (HypABCDEF). While the accessory protein HypB is associated with the insertion of Ni, HypCDEF proteins are responsible for the biosynthesis, assembly and insertion of the Fe(CN)_2_CO group (Watanabe et al. 2012). Among the three major classes (i.e. [Fe]-, [FeFe]-, and [NiFe]- hydrogenases), most known thermotolerant hydrogenases contain a [NiFe] active site and can be found within the domain of (aerobic and anaerobic) Bacteria and Archaea (Eberly and Ely 2008; Peters et al. 2015). Genome sequencing further showed the presence of genes associated with fermentation (lactate and mixed acid fermentation) and mannose metabolism, which is consistent with our data on utilized carbon sources (Table 1).

### Emended description of the genus *Thermoactinomyces* Tsiklinsky 1899 (Approved Lists 1980) emend. Yoon et al. (2005)

The description is as given by Yoon et al. (2005) with the following modifications. The genus includes aerobic as well as anaerobic members. Anaerobic fermentative metabolism involves the production of acids and hydrogen. The G + C content may range from 44.7 to 49.1 mol%. The genus includes a new species, *T. mirandus*.

### Description of *Thermoactinomyces mirandus* sp. nov.

*Thermoactinomyces mirandus* (mir.an’dus. L. masc. adj. (gerundive of L. v. *miror*), to be admired).

*Thermoactinomyces mirandus* forms a white branching, septate mycelium that aggregates into pellets in liquid culture. *T. mirandus* requires anaerobic conditions and grows well on lactose, lactose/acetate, mannose, and glucose, whereas growth on yeast and meat extract is poor. Small amendments of yeast extract; however, are mandatory for growth. A combination of lactose and acetate accelerates growth. Products from lactose fermentation are lactate, acetate, ethanol, H_2_ and CO_2_. Growth occurs in the range of 45–60 °C and pH 5.0–9.0, with optima at 55 °C and pH 7.0, respectively (Table 1). The optimal NaCl concentration is 0–2% (w/v). *T. mirandus* has a G + C content of 44.7 mol%.

The type strain, AMNI-1^T^ (= DSM 110094^T^ = LMG 31503^T^), was isolated from a thermophilic biogas plant in Roppen (Tyrol, Austria). The GenBank accession number is: MN148883.

## Data Availability

The GenBank accession number of strain AMNI-1^T^ is MN148883. The type strain AMNI-1^T^ can be obtained from the German Collection of Microorganisms and Cell Cultures GmbH (DSM 110094^T^) and the Belgian Co-ordinated Collections of Micro-organisms (LMG 31503^T^).
